# Systemic delivery of anti-sense oligonucleotide targeting a-synuclein for the treatment of multiple system atrophy

**DOI:** 10.21203/rs.3.rs-7908950/v1

**Published:** 2025-11-07

**Authors:** Brian Spencer, Bao Quach, Sahar Salehi, Robert A. Rissman

**Affiliations:** University of Southern California; University of Southern California; University of Southern California; University of Southern California

**Keywords:** Multiple System Atrophy, alpha Synuclein, transgenic mouse, systemic therapy, anti-sense oligonucleotides

## Abstract

Multiple System Atrophy (MSA) is a rare, sporadic, age-related synucleinopathy characterized by Parkinson-like motor symptoms and ataxia. There is no therapy for MSA other than symptomatic treatment. MSA is characterized pathologically by glial cytoplasmic inclusions (GCI) of a-synuclein (aSyn) occurring in oligodendrocytes leading to loss of myelination in the brain. We recently utilized a peptide-mediated delivery method to systemically transport an anti-sense oligonucleotide (ASO) targeted to aSyn in a mouse model of MSA. We hypothesized that systemic delivery of aSyn ASO by peptide mediated delivery to a mouse model of MSA would reduce the aSyn accumulation in oligodendrocytes and reduce the overt pathology associated with MSA. Following monthly treatments of the aSyn ASO, we found increased myelination in the corpus callosum and the cerebellum. We also observed increased numbers of oligodendrocytes and reduced gliosis; however, we did not detect changes in overall aSyn in the areas of the brain we examined. Upon further analysis, we determined the peptide-mediated delivery of aSyn ASO was not taken up by oligodendrocytes. Thus, we have successfully alleviated some of the pathology associated with MSA in a mouse model; however, without direct delivery to oligodendrocytes, other approaches may need to supplement this therapy.

## Introduction

Multiple System Atrophy (MSA) is a rare, sporadic, age-related synucleinopathy characterized by accumulation of a-synuclein (aSyn) in the form of glial cytoplasmic inclusions (GCI) in the oligodendrocytes of the central nervous system, leading to myelin loss, neuronal loss and neuronal inflammation. [26, 30]. MSA presents as two unique neurodegenerative diseases characterized by loss of neurons in the striatonigral regions leading to Parkinsonian-like motor symptoms like bradykinesia and postural instability (MSA-P) or cerebellar ataxia (MSA-C).

MSA-C is characterized by aSyn accumulation initially within the pontine cerebellar projections and cerebellar white matter, which is accompanied by pathological phosphorylation at Ser129 (P-Syn) [19]. To date it is controversial how oligodendrocytes accumulate pathological aSyn; however, clear evidence exists that oligodendrocyte dysfunction is characterized by accumulation of cytoplasmic inclusions containing aSyn and P-Syn which leads to myelin loss and ultimately to loss of neurons as well as gliosis (reviewed [3]). Currently there is no disease modifying therapy available for MSA, and even symptomatic treatment is limited, particularly for MSA-C.

Currently available transgenic mouse models of MSA over-express aSyn under oligodendrocyte specific promoters such as the proteolipid promoter [9] and the cyclic nucleotide phosphodiesterase promoter [33]. We characterized a transgenic mouse model of MSA-C (MBP29) developed by over-expressing human aSyn with the oligodendrocyte specific myelin basic protein promoter (MBP) resulting in accumulation of aSyn in GCI of oligodendrocytes [23]. This model expresses and accumulates aSyn in the cortex, basal ganglia, corpus callosum and the cerebellum [23, 28, 29]. Meszaros characterized the cerebellar accumulation of aSyn in the MBP29 mouse model of MSA and concluded that the model can best be described as a model of MSA-C based on myelin deficits in the cerebellar white matter, loss of Purkinje neurons in the cerebellum and motor deficits including decreased walking speed and gait instability [17]. All this is associated with aSyn accumulation in the oligodendrocytes suggesting the MBP29 mouse is a model of MSA-C.

Antisense oligonucleotide (ASO) therapy is an effective means for modifying expression of genes. Delivery of a short 20–30 nucleotide sequence anti-sense to the specific gene transcript in affected cells can reduce protein expression by either steric hinderance or activation of RNase and RNA degradation (reviewed [11]). ASOs and related splice switching oligonucleotides delivered for neurodegenerative diseases are either in clinical trials or in some cases approved therapeutics [4, 14, 15, 22]. However, ASO therapies for neurodegenerative disorders require intra-thecal administration because the ASO does not cross the blood-brain barrier (BBB). We have developed a peptide mediated transport approach for the delivery ASOs to the brain following systemic administration.

We identified an 11 amino acid peptide fragment derived from Apolipoprotein B (ApoB^11^) that binds to and is transported by the low-density lipoprotein receptor (LDL-R) [1, 2, 13, 24]. We have shown the ability of the ApoB peptide (ApoB^11^) to transport an ASO sequence targeted to aSyn for a systemic therapy for synucleinopathies that has proven effective in synucleinopathy models of Parkinson’s disease and Dementia with Lewy Bodies [12, 24].

To determine if systemic peptide mediated delivery of an aSyn ASO could be an effective therapy for the aSyn accumulation in oligodendrocytes of MSA, we delivered the ApoB^11^-aSyn ASO by intra-peritoneal injection monthly for 2 months to the MBP29 mouse model of MSA-C. We hypothesized that delivery to the mouse model of MSA-C, we would reduce the accumulation of aSyn in oligodendrocytes leading to increased myelination, reduced neuronal loss and reduced neuroinflammation. Our hope is to find new, relevant and long-lasting therapies for MSA patients.

## Materials and Methods

### Synuclein Transgenic Mice:

For this project, mice expressing the human aSyn under the control of the myelin basic protein promoter (MBP29) were used [23]. We used the MBP29 line as these animals express a high level of aSyn in oligodendrocytes in the corpus callosum, cortex, cerebellum and brainstem along with associated loss of myelin and neuroinflammation beginning at 2–3 months of age [7, 8, 17, 23].

Female, transgenic and non-transgenic littermates were used for this experiment as previous characterization of the MBP29 mouse model of MSA-C was performed only on female mice [7]. Mice received intra-peritoneal injections of the peptide-ASO complex at a dosage of 2 mg/kg (volume 10ml/kg) every 4 weeks for 8 weeks beginning at 3-months of age. Treatment dose and frequency were determined from previous successful ASO delivery and pharmacokinetic analysis [1, 2, 12, 24]. Treatment length was limited to 2 months as the average life span of the MBP29 mouse is 5–6 months. Treatment involved a synthetic peptide, ApoB^11^, conjugated with a 2′-O-methyl (2′-OMe) antisense oligonucleotide (ASO) we previously showed targeted human aSyn [12, 24]. ApoB^11^ peptide (NH_2_-RLTRKRGLKLAGGGGGRRRRRRRRR) was synthesized to 95% purity (GenScript) and resuspended in nuclease-free water. aSyn ASO (2’MOE 5’GAC TTT CAA AGG CCA AGG A) and a scrambled control (Scr) ASO (2’MOE 5’GGG CAT ACT GAG CTA ACA A) (Integrated DNA Technology) were synthesized, purified, and resuspended in RNase-free TE buffer. The ApoB^11^ peptide and the respective ASO were incubated in PBS to form peptide-ASO complexes at a ratio of 10:1 for 30 minutes at room temperature prior to injection [12]. At least 4 animals per group were treated, and were sacrificed four weeks after receiving the final treatment.

All experimental procedures involving animals were approved by the Institutional Animal Care and Use Committee of UCSD, following the NIH Guide for the Care and Use of Laboratory Animals (protocol #S02221). Efforts were made to minimize animal suffering and to reduce the number of animals used.

### Anatomical Studies:

After the completion of treatments, mice were euthanized in accordance with NIH guidelines followed by brain extraction. Right hemi-brains were immersion-fixed in 4% phosphate-buffered formaldehyde for 48 hours at 4°C and subsequently sectioned sagittally at 40 μm with a vibratome. The left hemi-brains were snap-frozen in liquid nitrogen for biochemical analysis.

For neuropathological examination, serial brain sections between 1.25 and 1.75 mm lateral to Bregma were immunolabeled overnight at 4°C with antibodies specific to total aSyn (BD Transduction Laboratories, Cat# 610787, RRID:AB_398108) phosphorylated αSyn (Ser129) (Abclonal, Cat# AP0450, RRID: AB_2771549), Olig2 (Novus Biologicals, Cat# NBP1–28667, RRID:AB_1914109), myelin basic protein (MBP, Abcam, Cat# ab218011, RRID:AB_2895537), NeuN (Millipore, Cat# MAB377, RRID:AB_2298772), GFAP (Millipore, Cat# MAB3402, RRID:AB_94844) and Iba1 (FUJIFILM Wako Pure Chemical Corporation, Cat# 019–19741, RRID:AB_839504). The sections were then incubated with biotinylated secondary antibodies (Vector Laboratories) and visualized using an avidin-biotin complex (ABC Elite; Vector Laboratories) followed by diaminobenzidine (DAB) staining. Slides were scanned using the NanoZoomer S60 Digital Slide Scanner (Hammamatsu, 20X). Regions of interest were isolated using NDP.View2 and analyzed with CellProfiler or ImageJ. For each group of animals, at least 4 brains were imaged and one image from each subregion was analyzed

Luxol Fast Blue (LFB) staining was performed as previously described [25, 27]. Slides were scanned using the NanoZoomer S60 Digital Slide Scanner and regions of interest were isolated using NDP.View2. For LFB and MBP staining, optical density was measured and normalized on each slide with optical density of the cortex. At least 3 different areas of the corpus callosum and brainstem and 2 areas each in the interpositis nuclei and inferior pedunculus of the cerebellum were measured. For each group of animals, at least 4 brains were analyzed.

### Double immunolabeling and fluorescence co-labeling:

To determine the co-localization of the systemically delivered aSyn ASO and individual cells or aSyn protein, we constructed a biotinylated aSyn ASO (Integrated DNA Technology). This aSyn ASO was the same sequence as delivered for the rest of the experiments with the addition of 5’-terminal biotin. 5-month-old female MBP-aSyn tg and non-tg mice (N=3 each) received a single intra-peritoneal injection of ApoB^11^-aSyn biotin-ASO at 4 mg/kg. Three days after injection, mice were sacrificed and perfused with ice cold PBS to clear blood from vessels and then 4% paraformaldehyde to fix the brain. Brains were sectioned on a vibratome at 40μm thickness and sections between 1.25 and 1.75 mm from Bregma were incubated overnight with Tyramide Signal Amplification Direct (red) (ThermoFisher) to label the biotinylated ASO and antibodies for Olig2 (Novus Biologicals), aSyn (BD Transduction Laboratories), NeuN (Millipore), or GFAP (Millipore) overnight at 4°C. Sections were then incubated with species appropriate secondary antibodies labeled with FITC (green) (Vector Laboratory) and mounted with ProLong Gold Antifade (Fisher Scientific). Sections were imaged by laser scanning confocal microscopy with a Leica Stellaris 8.

### Biochemical Analysis:

Frozen left hemi-brains from 4 mice per group were dissected into cortex, cerebellum, and brainstem regions. These regions were homogenized (1X nuclease free PBS, RNAse inhibitor (NEB), protease inhibitor (Mini-Complete, Roche)) with Bead Mill 24 (Fisher) using 1.4mm ceramic beads (Fisher). Following homogenization, samples were removed for protein and incubated with 10X RIPA. Protein was quantified by BCA assay (BioRad).

Immunoblotting was performed with 25μg protein per lane loaded in a 12% Bis-Tris SDS-PAGE gel (Criterion TGX, BioRad) and transferred onto PVDF membranes using semi-dry Trans-Blot Turbo Transfer System (BioRad). The membranes were probed with antibodies against total αSyn, phosphorylated aSyn, myelin basic protein, calbindin (Abcam, Cat# ab82812, RRID:AB_1658451), and GAPDH (Signaling Technology, Cat# 2118, RRID:AB_561053) followed by species appropriate HRP conjugated antibodies (BioRad). Proteins were visualized using enhanced chemiluminescence (SuperSignal West Pico PLUS, ThermoScientific), and quantification was performed using a ChemiDoc Imaging System (BioRad). Band intensities were normalized to GAPDH levels and compared to non-transgenic controls. Blots were stripped (Restore Western blot stripping buffer, ThermoScientific) and re-probed successively in the following order: P-Syn, T-Syn, MBP, and GAPDH and with a separate blot, MBP, calbindin and GAPDH.

RNA was extracted from the remaining homogenized sample (N=4 for each group of animals) using RNAeasy kit (Qiagen) and quantified by spectrophotometry readings. For cDNA synthesis, 500ng of total RNA was reverse transcribed using iScript gDNA Clear cDNA Synthesis kit (BioRad). Real Time-PCR (RT-PCR) was performed using IDT PrimeTime Gene Expression Master Mix (IDT) with primer/ probe designed for mouse myelin basic protein (IDT, Mm.PT.58.28532164), calbindin (IDT, Mm.PT.58.29798692) and human aSyn (IDT, Hs.PT.58.912923). Samples were analyzed with a real time PCR system (CFX96 Real Time System, BioRad). The amount of cDNA was calculated by the comparative threshold cycle method and expressed using mouse actin (Mm.PT.39a.22214843.g; IDT) as an internal control and then this was normalized to wild type mice treated with ApoB^11^:Scr ASO.

### Statistical Analysis:

All statistical analyses were conducted using GraphPad Prism v10.0. Group comparisons were made using two-way ANOVA with Tukey’s test among groups. Significance was set at p < 0.05. Results are expressed as mean ± standard error of the mean (SEM).

## Results

**Figure 1:**
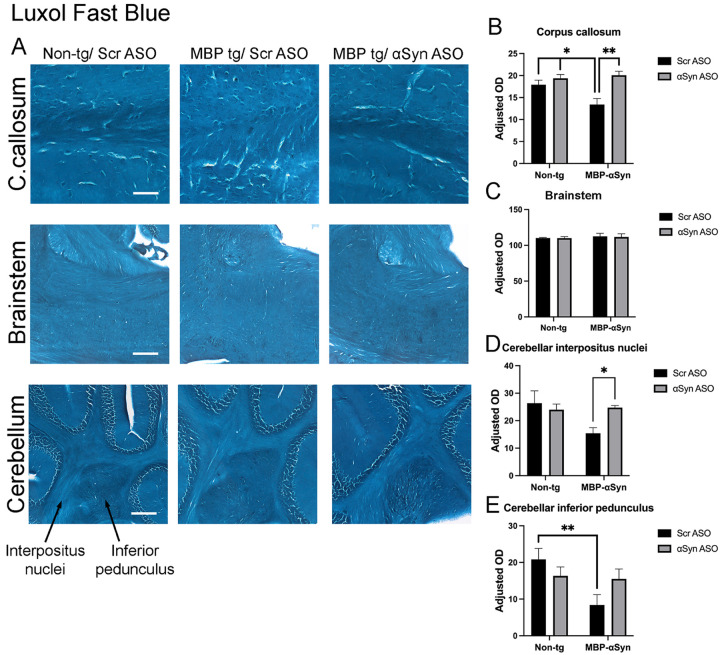
Luxol Fast Blue (LFB) staining in MBP-aSyn tg and non-tg mice treated with ApoB^11^-Scr ASO and aSyn ASO. (A) Representative immunohistochemistry images showing LFB histochemistry staining in various brain regions, including the corpus callosum (C.callosum), brainstem and cerebellum. Scale bars = 100μm for C.callosum and brainstem; 200μm for cerebellum. Quantification of MBP adjusted optical density is an average of 2 locations in the (B) C.callosum, (C) brainstem, (D) interpositus nuclei and (D) inferior pedunculus of the cerebellum in non-tg and MBP-aSyn-tg mice treated with Scr ASO or aSyn ASO corrected for background staining in the cortex. Arrows indicate location of the interpositus nuclei and inferior pedunculus in the cerebellar white matter. Data represent mean ± SEM, * p< 0.05, ** p<0.01. At least 4 mice per group were analyzed. Statistical analysis was conducted with two-way ANOVA (mixed model) with Tukey’s test among groups.

*aSyn ASO prevents reduced myelination seen in MBP-aSyn tg mice*. Oligodendrocytes are responsible for myelination of neurons and MSA patients show reduced myelination associated with aSyn accumulation in oligodendrocytes [17]. Previous characterization of the MBP-aSyn tg line 29 mice has shown reduced myelination in the corpus callous and the cerebellum particularly in the interpositus nuclei and the inferior pedunculus [7, 8, 17, 23, 25]. We stained sections from the mice with Luxol fast blue (LFB) which binds to myelin and indeed, MBP-aSyn tg mice had reduced LFB staining in the corpus callosum and areas of the cerebellum as previously reported indicating a loss of myelin; however, no loss of LFB staining was observed in the brainstem (Figure 1A). Immunohistochemistry for MBP protein showed reductions in the corpus callosum, brainstem and cerebellum (Supplemental Figure 1A). Treatment of the MBP-aSyn tg mice with the ApoB^11^-aSyn ASO resulted in significantly increased LFB staining in the corpus callosum and the cerebellar interpositus nuclei comparable to wild type mouse levels (Fig 1B–D). We also observed an increase in LFB staining in the cerebellar inferior pedunculus although this was not significant (Fig 1E). Similar results were observed with MBP immunohistochemistry with restored levels of MBP in corpus callosum (Supplemental Figure 1B). MBP IHC also showed reduced protein levels in the brainstem and cerebellar inferior pedunculus that was increased by treatment with ApoB^11^-aSyn ASO although not to levels measured in non-tg mice (Supplemental Figure 1C-E).

**Figure 2:**
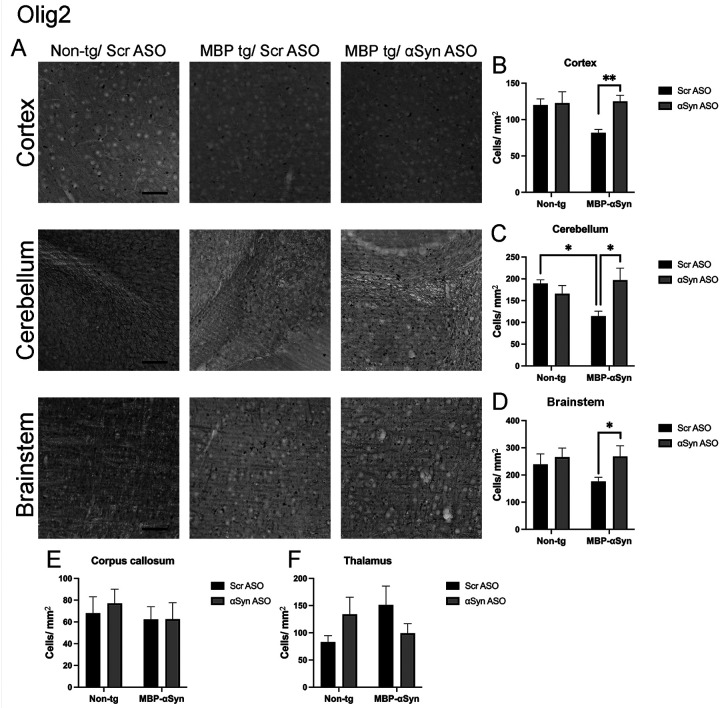
Olig2 staining in MBP-aSyn tg and non-tg mice treated with ApoB^11^-Scr ASO and aSyn ASO. (A) Representative immunohistochemistry images showing Olig2 staining in various brain regions, including the cortex, cerebellum and brainstem. Scale bars = 100μm. Quantification of Olig2 positive cells/ μm^2^ in the (B) cortex, (C) cerebellum, (D) brainstem, (E) corpus callosum and (F) thalamus in non-tg and MBPaSyn-tg mice treated with Scr ASO or aSyn ASO. Data represent mean ± SEM, * p< 0.05, ** p<0.01. At least 4 mice per group were analyzed. Statistical analysis was conducted with two-way ANOVA (mixed model) with Tukey’s test among groups.

MBP-aSyn tg mice have been shown to have reduced number of oligodendrocytes in the cortex, brainstem and corpus callosum at 5 months of age despite elevated numbers at younger ages [7, 17]. Similarly, we observed reduced oligodendrocytes in the cortex, cerebellum and brainstem as determined by immunohistochemistry with the Olig2 antibody at 5 months of age (Figure 2A–D). We did not observe a change in the number of oligodendrocytes in the corpus callosum or thalamus (Figure 2E,F). Treatment with ApoB^11^-aSyn ASO restored the number of oligodendrocytes in the cortex, cerebellum and brainstem to non-tg levels (Figure 2B,C,D). Thus, systemic delivery of ApoB^11^-aSyn ASO prevented the loss of oligodendrocytes and myelination in the MBP-aSyn tg mice.

### Treatment with aSyn ASO and accumulation of aSyn and PSyn

**Figure 3:**
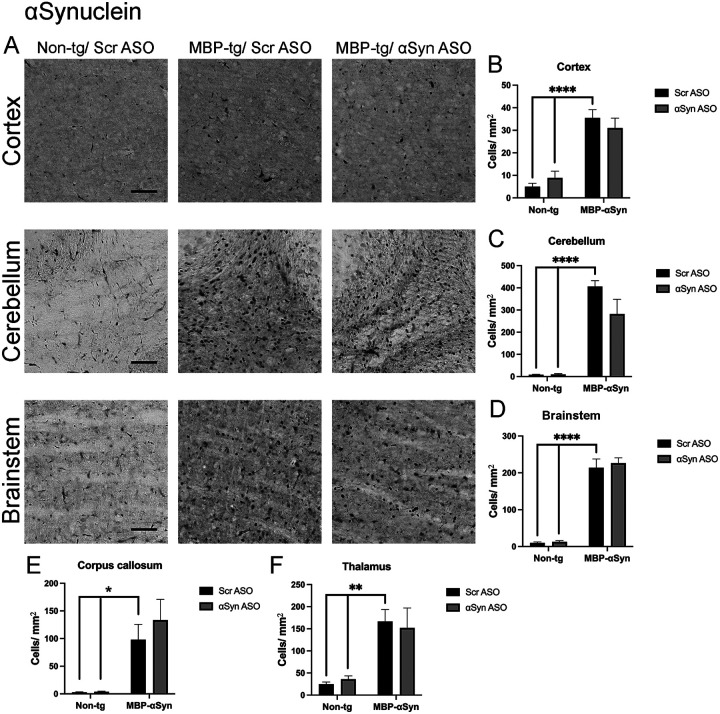
aSyn accumulation in MBP-aSyn tg (MBP-tg) and non-tg mice treated with ApoB^11^-Scr ASO and aSyn ASO. (A) Representative immunohistochemistry images showing aSyn staining in various brain regions, including the cortex, cerebellum and brainstem. Scale bars = 100μm. Quantification of aSyn positive cells/ μm^2^ in the (B) cortex, (C) cerebellum, (D) brainstem, (E) corpus callosum and (D) thalamus in non-tg and MBP-aSyn-tg mice treated with Scr ASO or aSyn ASO. Data represent mean ± SEM, * p< 0.05, ** p<0.01, **** p<0.0001. At least 4 mice per group were analyzed. Statistical analysis was conducted with two-way ANOVA (mixed model) with Tukey’s test among groups.

MBP29-aSyn tg mice overexpress and accumulate human aSyn in oligodendrocytes throughout the CNS but particularly in the cortex, corpus callosum, cerebellum and brainstem [23]. To determine if an ASO targeted to aSyn could reduce the oligodendrocyte accumulation of aSyn, we systemically delivered ApoB^11^-aSyn ASO to transgenic and non-transgenic mice. As previously reported for MBP-aSyn tg mice, we observed aSyn accumulation in the cortex, corpus callosum and cerebellum (Fig 3A,B,C,E). We also examined the brainstem and thalamus where aSyn accumulated in small punctate locations (Fig 3A,D,F). Delivery of the ApoB^11^-aSyn ASO did not significantly affect the number of cells accumulating aSyn in any region examined although a slight reduction in the number of cells accumulating aSyn in the cerebellum was observed (Fig 3C).

**Figure 4:**
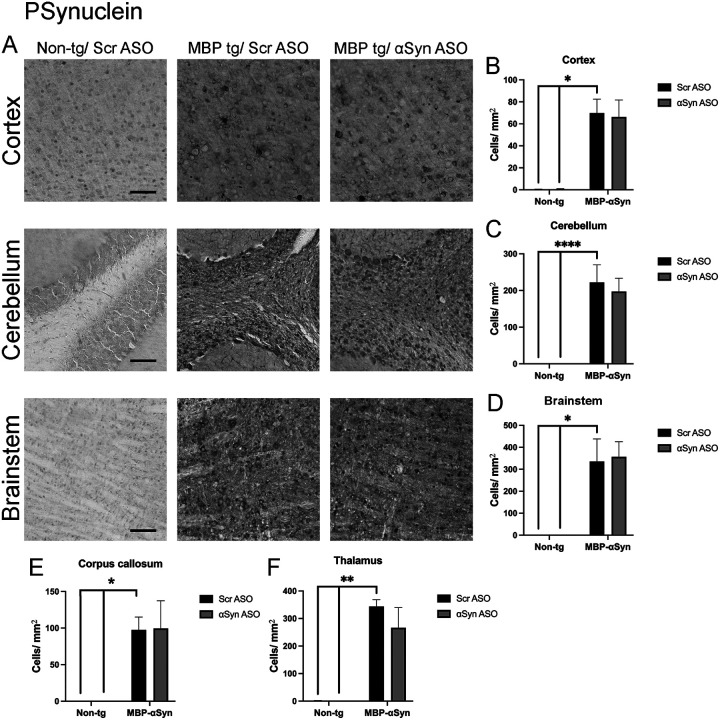
Phosphorylated aSyn (Ser129) (PSyn) accumulation in MBP-aSyn tg (MBP-tg) and non-tg mice treated with ApoB^11^-Scr ASO and aSyn ASO. (A) Representative immunohistochemistry images showing PSyn staining in various brain regions, including the cortex, cerebellum and brainstem. Scale bars = 100μm. Quantification of PSyn positive cells/ μm^2^ in the (B) cortex, (C) cerebellum, (D) brainstem, (E) corpus callosum and (F) thalamus in wild type and MBP-aSyn-tg mice treated with Scr ASO or aSyn ASO. Data represent mean ± SEM, * p< 0.05, ** p<0.01, **** p<0.0001. At least 4 mice per group were analyzed. Statistical analysis was conducted with two-way ANOVA (mixed model) with Tukey’s test among groups.

Phosphorylation of aSyn at Ser129 (PSyn) has been identified in GCI and has been linked to aSyn aggregation and neuronal loss [19]. Mouse brain sections were stained for the Ser129 PSyn. As previously reported, we observed significant accumulation of PSyn in the MBP-aSyn tg mice in the cortex, cerebellum and corpus callosum (Fig 4A,B,C,E). We extended this study to examine the brainstem and thalamus where we also observed significant PSyn accumulation (Fig 4A,D,F). Treatment of the MBP-aSyn tg mice with the ApoB^11^-aSyn ASO did affect the number of cells with accumulated PSyn in any region examined.

### aSyn ASO impact on gliosis

**Figure 5:**
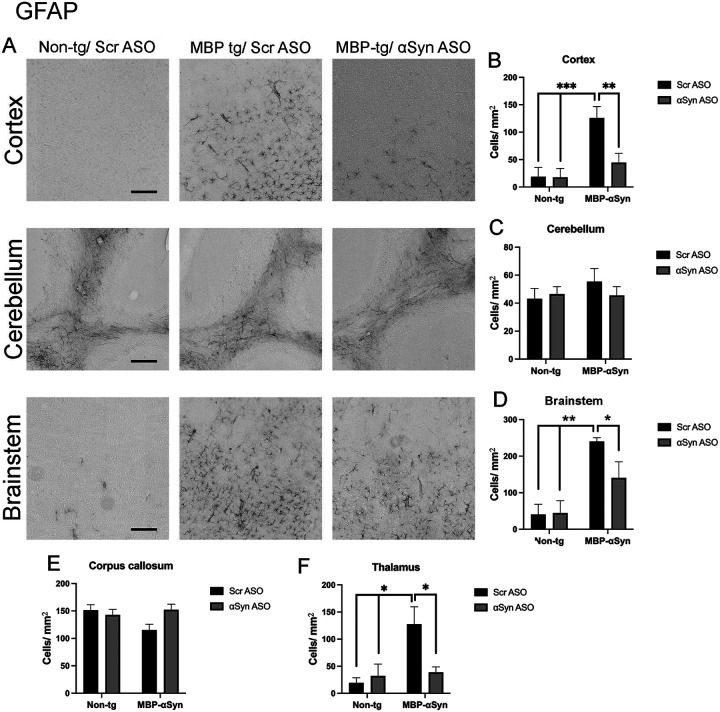
GFAP staining in MBP-aSyn tg and non-tg mice treated with ApoB^11^-Scr ASO and aSyn ASO. (A) Representative immunohistochemistry images showing GFAP staining in various brain regions, including the cortex, cerebellum and brainstem. Scale bars = 200μm. Quantification of GFAP positive cells/ μm^2^ in the (B) cortex, (C) cerebellum, (D) brainstem, (E) corpus callosum and (F) thalamus in non-tg and MBP-aSyn-tg mice treated with Scr ASO or aSyn ASO. Data represent mean ± SEM, * p< 0.05, ** p<0.01, *** p<0.001. At least 4 mice per group were analyzed. Statistical analysis was conducted with two-way ANOVA (mixed model) with Tukey’s test among groups.

Increased gliosis has been shown in mouse models of synucleinopathies as well as post-mortem in patients with PD and MSA [8, 10, 29]. Indeed, the MBP-aSyn mouse model of MSA has elevated astrocytosis in the cortex and corpus callosum and increased microglia [8, 17, 23]. We confirmed these findings showing elevated astrocytosis in the cortex, brainstem and thalamus in MBP-aSyn tg mice (Figure 5A,B,D,F). treatment with the ApoB^11^-aSyn ASO reduced the number of astrocytes in the cortex, brainstem and thalamus to levels observed in non-tg littermates. We did not observe increased astrocytosis in the cerebellum or corpus callosum (Figure 5A,C,E).

**Figure 6:**
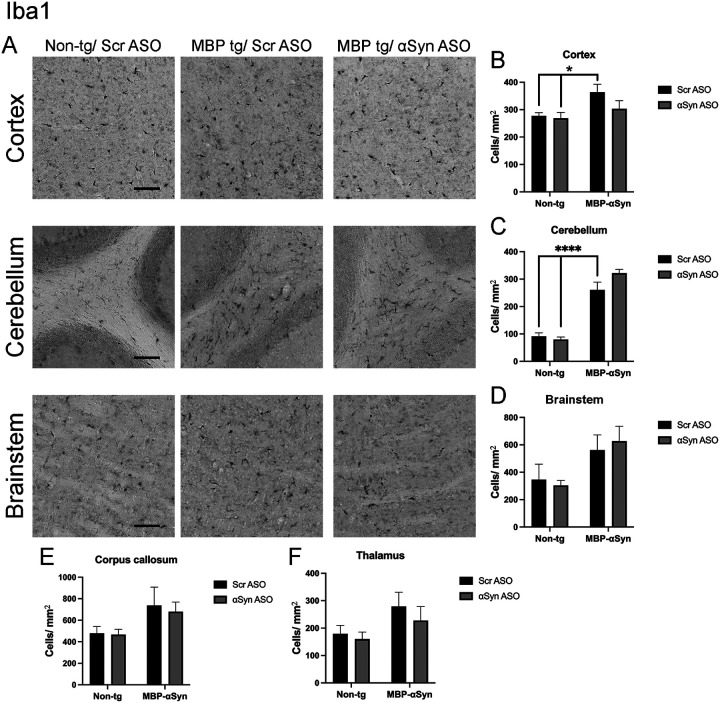
Iba1 staining in MBP-aSyn tg and non-tg mice treated with ApoB^11^-Scr ASO and aSyn ASO. (A) Representative immunohistochemistry images showing Iba1 staining in various brain regions, including the cortex, cerebellum and brainstem. Scale bars = 100μm. Quantification of Iba1 positive cells/ μm^2^ in the (B) cortex, (C) cerebellum, (D) brainstem, (E) corpus callosum and (F) thalamus in non-tg and MBP-aSyn-tg mice treated with Scr ASO or aSyn ASO. Data represent mean ± SEM, * p< 0.05, **** p<0.0001. At least 4 mice per group were analyzed. Statistical analysis was conducted with two-way ANOVA (mixed model) with Tukey’s test among groups.

Similarly, we observed increased number of microglia in the cortex, cerebellum, brainstem, corpus callosum and thalamus of MBP-aSyn tg mice (Figure 6A–F). In contrast to the reduction of astrocytes, we did not measure a significant change in microglial activation following treatment with the aSyn ASO, suggesting the treatment effect was limited to astrocytes.

**Figure 7:**
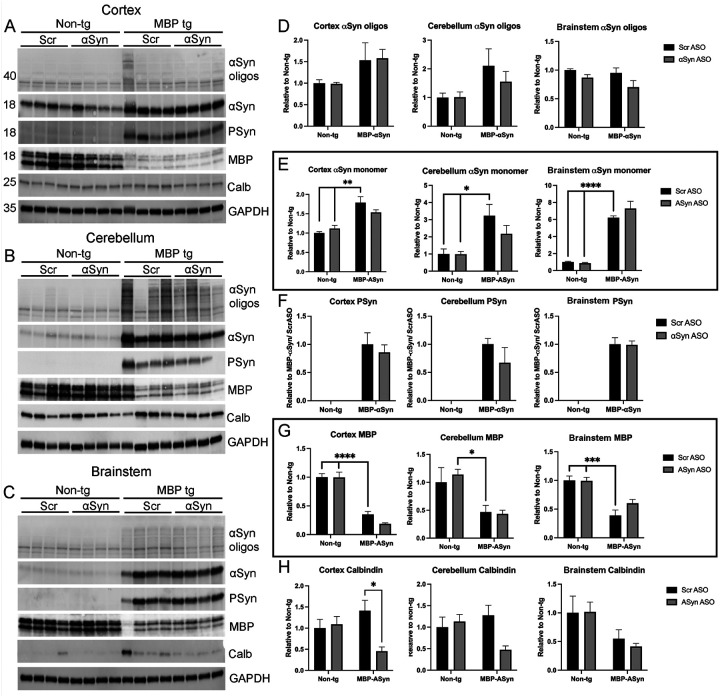
Immunoblot analysis of aSyn, PSyn, myelin basic protein (MBP) and calbindin (Calb) in MBP-aSyn tg and non-tg mice treated with ApoB^11^-Scr ASO or aSyn ASO. Frozen hemi-brains were sub-dissected to isolate cortex, cerebellum and brainstem regions. Representative immunoblots from (A) cortex, (B) cerebellum and (C) brainstem enriched regions analyzed with antibodies specific for aSyn monomers (18kDa) and oligomers (>36kDa), P-aSyn (18kDa), MBP (14–20kDa), Calb (25kDa) and GAPDH (35kDa). Graphs represent quantitation of immunoblots showing relative levels of (D) oligomeric aSyn, (E) monomeric aSyn, (F) PSyn, (G) MBP, and (H) Calbindin) protein normalized to GAPDH and then normalized to wild type treated with ApoB^11^-Scr ASO except PSyn which was normalized to MBP-aSyn tg mice treated with ApoB^11^-Scr ASO. Data represent mean ± SEM, * p<0.05, ** p<0.01, *** p<0.001, **** p<0.00001. N=4 mice per group. Statistical analysis was conducted with two-ANOVA (mixed model) with Tukey’s test among groups.

**Figure 8:**
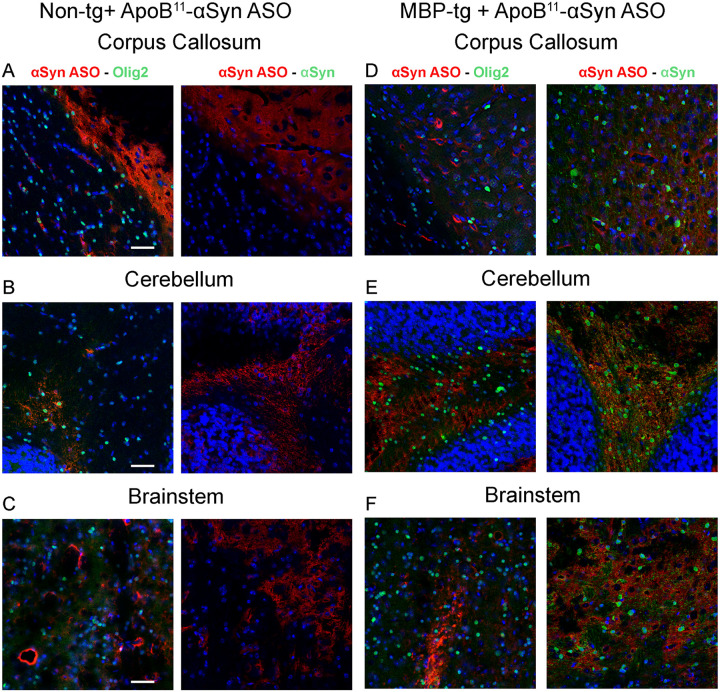
Co-localization of biotinylated aSyn ASO with oligodendrocytes (Olig2) and aSyn. Representative regions double labeled for aSyn ASO (red) and oligodendrocyte (Olig2, green) or aSyn (green) in (A,B,C) non-tg and (D,E,F) MBP-aSyn tg mice treated with ApoB^11^-aSyn ASO (biotinylated) in (A,D) corpus callosum, (B,E) cerebellum and (C,F) brainstem imaged with a LSCM. Scale bars = 100μm. N = 3 mice per group.

To confirm the IHC effects observed, we homogenized brain regions enriched in the cortex, cerebellum and brainstem for immunoblot analysis (Figure 7A–C) and qPCR for gene expression (Supplemental Figure 2). Oligomeric aSyn (Figure 7D), monomeric aSyn (Figure 7E), and Ser129 phosphorylated aSyn (PSyn) (Figure 7E) were detected at elevated levels in the MBP-aSyn tg mice compared to non-tg mice as expected. Similar to results from IHC, we did not measure changes in the accumulation of aSyn or PSyn in any of the brain regions analyzed following treatment with the ApoB-aSyn ASO. aSyn RNA levels were elevated in the MBP-aSyn tg mice compared to wild type; however, no change was detected following delivery of ApoB^11^-aSyn ASO (Supplemental Fig 2). We next assessed levels of myelin basic protein (MBP) in each of the same brain regions. Similar to results from LFB histochemistry, we measured significantly reduced levels of MBP in all three brain regions analyzed similar to previous reports [17, 23]; however, treatment with ApoB^11^-aSyn ASO did not affect the levels of MBP protein (Figure 8G). Interestingly, RNA levels of MBP appeared to be unchanged between MBP-aSyn tg and wild type mice (Supplemental Figure 2). Previous reports have shown reduced levels of calbindin protein in the cerebellum in the MBP aSyn tg mice at 3 months of age [17]. We observed no statistically significant change in calbindin protein levels in the cortex, cerebellum and brainstem in MBP aSyn tg mice compared to non-tg. Treatment with ApoB^11^-aSyn ASO caused a significant reduction in calbindin protein levels in the cortex only (Figure 7A,H). Similar results were observed when measuring the calbindin RNA by qPCR (Supplemental Figure 2).

### ApoB^11^-aSyn ASO does not colocalize with oligodendrocytes or aSyn

With little to no change in aSyn and PSyn by IHC, immunoblot or qPCR analysis, we examined whether the ApoB^11^-aSyn ASO was accumulating in affected oligodendrocytes following systemic intra-peritoneal delivery. Biotin labeled aSyn ASO was conjugated to the ApoB^11^ peptide as in earlier experiments and delivered intra-peritoneally to 5-month-old MBP-aSyn tg and non-tg littermate mice. Three days later mice were sacrificed, and brains were analyzed by IHC for the co-localization of biotin labeled aSyn ASO and either Olig2 (oligodendrocytes) or aSyn (Figure 8). Olig2 staining revealed abundant oligodendrocytes in the corpus callosum, cerebellum and brainstem in non-tg mice with fewer numbers in MBP-aSyn tg mice similar to results described earlier (Figure 8D–F). Biotin labeled aSyn ASO did not co-localize with the oligodendrocytes despite abundant staining in nearby cells that appeared to be neuronal or endothelial (Figure 8A–F). Similarly, although the aSyn was observed in the corpus callosum, cerebellum and brainstem of the MBP-aSyn tg mice, there was little to no co-localization with the biotin labeled aSyn ASO following systemic delivery (Figure 8D–F). In contrast, biotinylated ASO co-localized with both neurons (NeuN) and astrocytes (GFAP) in the corpus callosum, cerebellum and brainstem in both MBP-aSyn tg and non-tg mice (Supplemental Figure 3A–F) Thus, although systemic delivery of aSyn ASO with the ApoB^11^ peptide did cross the BBB and enter the CNS, it did not reach the target cell (oligodendrocyte) in the MBP-aSyn tg mouse model of MSA-C.

## Discussion

We investigated the systemic delivery of an ASO directed at aSyn for the treatment of MSA neuropathological changes. Treatment of MBP-aSyn tg mice with ASO reduced gliosis and increased both oligodendrocyte numbers as well as myelination without any measurable impact on accumulation of aSyn. Although ApoB^11^ peptide delivered the aSyn to the CNS, analysis showed little uptake by oligodendrocytes with uptake instead by surrounding neurons and astrocytes. This suggests a possible bystander affect whereby reducing aSyn in neurons and/or astrocytes has a beneficial effect on oligodendrocytes. It is worth mentioning that the aSyn ASO targets not only the human transgene expressed in oligodendrocytes but also the endogenous mouse aSyn that would be expressed in neurons [12, 24].

We have previously shown delivery of ASO or proteins with the ApoB LDLR binding domain to 20–30% of neurons and to 10–20% of glial cells across the whole brain; however, delivery to oligodendrocytes occurs at a significantly lower rate of ~ 1–2% [24, 25]. Indeed, we observed that the expression pattern of LDLR reflected the uptake and accumulation pattern in CNS cells in a mouse model of synucleinopathy [25]. These studies were conducted with a larger peptide also derived from the ApoB protein and involved the delivery of enzymes and antibodies. It was not known how the ApoB^11^ peptide conjugated with an ASO would distribute in the brain. We show here that the ApoB^11^ peptide successfully delivers an ASO to neurons and astrocytes (Supplemental Fig. 3); however, there is little to no delivery to oligodendrocytes (Fig. 8). Reports suggest that oligodendrocytes have LDLR and VLDLR expression for uptake of locally synthesized cholesterol to be integrated in myelin production [21, 34]; however, hypoxic ischemia resulted in reduced expression LDLR in oligodendrocytes in a mouse [32]. This suggests that pathological stimuli may affect LDLR expression in oligodendrocytes and this might suggest reduced LDLR expression in mouse models of synucleinopathies.

Treatment of the MBP-aSyn tg mouse model of MSA-C with ApoB^11^-aSyn ASO did not appear to reduce the accumulation of aSyn or PSyn by IHC or immunoblot. Despite this, we did measure increased myelination, and an increase proliferation of oligodendrocytes coupled with decreased gliosis. This suggests that the treatment had some effect on the MSA pathology. In view of the low level delivery of ASO to oligodendrocytes, there may have been a small reduction in aSyn accumulation as observed in the cerebellum (Fig. 4,8). In this view, even a small change in aSyn accumulation in oligodendrocytes may be enough to cause a positive outcome in MSA. Alternatively, since the aSyn ASO cross reacts with the endogenous mouse aSyn expressed in neurons, it may be that reducing aSyn in neurons induces uptake of aSyn from neighboring cells (i.e. oligodendrocytes) thus leading to a homeostasis of reduced human aSyn accumulation in GCI. This would not be reflected in the cell counts as the number of oligodendrocytes with accumulated αSyn would remain the same and only the amount of αSyn would be reduced.

Studies have shown cell to cell propagation of aSyn by extracellular endocytosis [5], endocytic vesicular transport [18] and even nanotubes [6]. In fact, we have observed therapeutic reduction in oligodendrocyte aSyn accumulation following systemic delivery of neurosin, an aSyn degrading enzyme, or an anti-aSyn antibody using the ApoB peptide delivery method [25, 29]. This suggests that targeting aSyn outside of oligodendrocytes can meaningfully impact aSyn GCI without directly targeting expression of the aSyn RNA transcript as attempted in this study.

While pharmacokinetics was not performed during this study, a recent study completed by us suggests that systemic administration of ApoB^11^ delivered ASO reaches the brain within 1 hour of injection with a half-life of approximately 28 days [2]. This aligns with previous investigation of a ApoB^11^ delivery of an siRNA to a mouse model of PD where we performed repeated administration every 30 days [12, 24]. For this study we utilized the MBP29 strain of the MBP-aSyn tg mouse which expressed high levels of aSyn with accumulation in the cortex, corpus callosum and cerebellum. However, longer term studies with a model expressing lower levels of aSyn might elicit more promising results. Indeed, the MBP1 strain of the same mouse line expresses significantly lower levels of aSyn (~ 20% less) and has a longer expected life span (~ 18 months) compared to 5–6 months for the MBP29 line [23] and might be better suited to long term delivery and therapeutic benefit described here.

It is not clear if the GCIs observed post-mortem in MSA originate in oligodendrocytes or are the result of uptake of aSyn from neurons (reviewed [3]). The MBP-aSyn tg mouse model of MSA recapitulates the accumulation of aSyn in the oligodendrocytes by overexpressing aSyn specifically in that cell. However, it has been suggested, aSyn protein originates from the neurons and is endocytosed by oligodendrocytes or oligodendrocyte precursor cells ultimately forming GCI [20, 31]. In fact, aSyn is present in oligodendrocyte precursor cells in post-mortem MSA patient brains and a mouse model of MSA [16]. Thus, targeting a therapy to reduce the expression of aSyn at the RNA level to oligodendrocytes might not be effective, and the MBP-aSyn tg mouse might not be the best model of full MSA pathological progression for this form of therapy intervention.

The MBP29 aSyn tg mice show elevated gliosis consisting of increased microglial and astrocyte cells in the grey matter similar to MSA-C patients [8, 23]. We also observed increased gliosis (astrocytes) in the brainstem as well as the cortex and the thalamus (Fig. 5). Similarly, we observed increased microglia in the cortex, cerebellum and to a lesser extent the brainstem and the thalamus of the MBP29 mice (Fig. 6). Thus, although there was widespread gliosis in the MBP29 mice, the exact cell type involved was different depending on the region of the brain with overall increases in microglia and astrocytes in cortex and brainstem. Treatment with the aSyn ASO reduced the astrocyte cell numbers associated with the MBP29 mice to levels comparable in non-tg mice; however, there was no effect on the microglial numbers.

Previous characterization of the MBP29 strain of MBP-aSyn tg mice showed elevated levels of oligodendrocytes in the corpus callosum and cerebellum accompanied by myelin loss at 3–4 months of age [7, 17]. We found decreased numbers of oligodendrocytes in the cortex, cerebellum and brainstem and no change in the corpus callosum at 5 months of age (Fig. 2). It is possible that increased GCI in the oligodendrocytes, while in the short term might result in proliferation, in the long term (~ 5 months of age) leads to loss of cells as a result of toxicity. In fact, increased apoptosis in oligodendrocytes at 9 months has been observed in a slower progressing MBP-aSyn tg mouse line (Line 1) [16]. Treatment with the ApoB^11^-aSyn ASO restored the oligodendrocytes or at least prevented their loss at 5 months of age. We confirmed previous findings of myelin loss and reduction in MBP protein [7, 16]; however, treatment with ApoB^11^-aSyn ASO prevented the loss of myelin so that myelination appeared similar to age-matched non-tg littermates.

## Conclusions

Systemic delivery of aSyn ASO through BBB transport with the peptide ApoB^11^ successfully increased myelination in the corpus callosum and the cerebellum and increased numbers of oligodendrocytes while reducing gliosis and providing therapeutic benefit in MBP-aSyn tg mice. However, lack of delivery of the aSyn ASO to oligodendrocytes may have tempered the overall effect of this therapeutic approach as no change in overall aSyn or PSyn was measured in any of the brain regions examined. Thus, the use of the ApoB^11^ peptide for delivery of ASO to oligodendrocytes for therapeutic approaches may not be the best approach for treatment of MSA.

## Supplementary Material

Supplementary Files

This is a list of supplementary files associated with this preprint. Click to download.

- SuppFig1MSAASO.tif
- SuppFig2MSAASO.jpg
- SuppFig3MSAASO.tif
- Uncroppedimmunoblotscortex.docx

## Data Availability

The datasets used and/or analyzed during the current study are available from the corresponding author upon reasonable request.

## Abbreviations

aSyn: Alpha synuclein
MSA: Multiple system atrophy
GCI: Glial cytoplasmic inclusions
ASO: Anti-sense oligonucleotide
MSA-C: Multiple system atrophy with cerebellar ataxia symptoms
MSA-P: Multiple system atrophy with Parkinsonian symptoms
MBP: Myelin Basic Protein
ApoB: Apolipoprotein B
LDL-R: Low-density lipoprotein receptor
PSyn: Phospho Ser129 aSynuclein
TSyn: Total aSynuclein
LFB: Luxol fast blue
IHC: Immunohistochemistry
